# Characterization and Identification of Cryptic Biopeptides in* Carya illinoinensis* (Wangenh K. Koch) Storage Proteins

**DOI:** 10.1155/2017/1549156

**Published:** 2017-11-27

**Authors:** Everardo Mares-Mares, Santiago Gutiérrez-Vargas, Luis Pérez-Moreno, Leandro G. Ordoñez-Acevedo, José E. Barboza-Corona, Ma. Fabiola León-Galván

**Affiliations:** ^1^Posgrado en Biociencias, División de Ciencias de la Vida, Universidad de Guanajuato, Campus Irapuato-Salamanca, 36500 Irapuato, GTO, Mexico; ^2^Posgrado en Ciencias en Ingenieria Química, División de Ciencias Naturales y Exactas, Universidad de Guanajuato, Campus Guanajuato, 36000 Guanajuato, GTO, Mexico; ^3^Departamento de Agronomía, División de Ciencias de la Vida, Universidad de Guanajuato, Campus Irapuato-Salamanca, 36500 Irapuato, GTO, Mexico; ^4^Centro de Investigación y de Estudios Avanzados del IPN, Irapuato, Departamento de Biotecnología y Bioquímica, Libramiento Norte Carretera Irapuato-León Km 9.6, 36821 Irapuato, GTO, Mexico; ^5^Departamento de Alimentos, División de Ciencias de la Vida, Universidad de Guanajuato, Campus Irapuato-Salamanca, 36500 Irapuato, GTO, Mexico

## Abstract

The objective of this research was to identify and characterize the encoded peptides present in nut storage proteins of* Carya illinoinensis*. It was found, through in silico prediction, proteomic analysis, and MS spectrometry, that bioactive peptides were mainly found in albumin and glutelin fractions. Glutelin was the major fraction with ~53% of the nut storage proteins containing at least 21 peptides with different putative biological activities, including antihypertensives, antioxidants, immunomodulators, protease inhibitors, and inhibitors of cell cycle progression in cancer cells. Data showed that using 50 *μ*g/mL tryptic digests of enriched peptides obtained from nut glutelins is able to induce up to 19% of apoptosis in both HeLa and CasKi cervical cancer cells. To our knowledge, this is the first report that shows the potential value of the nut-encoded peptides to be considered as adjuvants in cancer therapies.

## 1. Introduction

Pecan is a fruit with a single stone surrounded by a husk, native of United States and México, but it has a worldwide distribution. The interest for studying Pecan has been incremented in the last years, mainly because its nut is considered as a healthy foodstuff. In this regard, it has been observed that its regular consumption decreases the risk to suffer coronary heart disease and type 2 diabetes [1]. Nut is a high-protein food as it contains from 18 to 24% on a dry weight basis [2]. When proteins from meat and plants are ingested, they elicit a wide range of nutritional and biological properties, some of which are attributed to the presence of encrypted bioactive peptides [3]. Several reports indicate that storage proteins from plants, such as soy, amaranth, and wheat, are a source of bioactive peptides [4, 5]. These peptides are inactive within the sequence of parent proteins, but they can be released during gastrointestinal digestion or foods processing [6]. Depending on the amino acid sequence, these peptides may exert a number of different activities, for example, as antihypertensive, antioxidant, antiproliferative (anticarcinogenic) activity, antithrombotic, opioid, inhibitor of enzymes, activator of proteolysis, stimulant, and metabolic regulator. Cytochemical studies have provided evidences that food-derived bioactive peptides modulate viability, proliferation, differentiation, and apoptosis of different cell types [7], and also they are able of inhibiting cancer cells [8, 9].

In Mexico, the nut is called “the queen of dried fruits,” because of its nutritional importance. It is ingested alone or mixed with other dried fruits, in desserts and ice creams, among others. Although nut has been studied in different aspects [1], to our knowledge there are no reports about either the presence of encrypted bioactive peptides in this seed, nor its role against cancer cells. In this work, we obtained different Pecan nut's protein fractions and small peptides by first time, assigned putative physiological roles, and demonstrated its effect on HeLa and CasKi cells from cervical cancer.

## 2. Materials and Methods

### 2.1. Protein Isolation of Nut

Seeds (nut) of* Carya illinoinensis* (Wangenh K. Koch), Wichita variety, were degreased for 7 h in petroleum ether, at a ratio nut/petroleum ether 1 : 14 (w/v) with the Soxhlet method reported by the AOAC [17]. The total protein isolation was developed according to the protocol established by Saravanan and Rose [18]. In brief, 5 g of nut flour was mixed with 15 mL of cold acetone containing 10% trichloroacetic acid and 0.07%  *β*-mercaptoethanol. The mixture was homogenized by sonication for 15 min on ice and centrifuged at 2,000 ×g for 2 minutes at 4°C and proteins in the supernatant were precipitated overnight at −20°C. Proteins were concentrated by centrifugation at 10,000 ×g for 30 min at 4°C; the pellet was washed 3x in cold acetone and then dried at room temperature to remove excess of acetone and stored at −20°C [18]. For storage protein isolation, albumin fraction with nonprotein nitrogen (NPN) was obtained using distilled water as extraction agent. The suspensions of flour/solvent (1 : 10 w/v) were extracted with magnetic stirring for 1 h at 4°C and centrifuged at 10,000 ×g for 15 min at 4°C. The supernatant was collected and stored at −20°C for subsequent analysis. Pellet was resuspended in 0.1 M NaCl, 0.010 M K_2_HPO_4_ (pH 7.5), and 0.001 M EDTA for extracting 7S globulins. The 11S globulin fraction was obtained with 0.8 M NaCl, 0.010 K_2_HPO4, and 0.001 M EDTA (pH 7.5) according to the report by De La Barba Rosa et al. [19]. Prolamin extraction was carried out using four direct extraction solvents: 70% ethanol [19]; 70% ethanol and 0.5% sodium acetate [20]; 70% ethanol, 0.5% sodium acetate, and 1%  *β*-mercaptoethanol [21]; and 60% 2-propanol with 1%  *β*-mercaptoethanol [22]. Finally, glutelins were obtained with a 0.1 M NaOH solution [19]. Protein fractions were quantified in triplicate using the Bradford method (Bio-Rad, Hercules, CA, USA) and then analyzed by Sodium Dodecyl Sulfate-Polyacrylamide Gel Electrophoresis (SDS-PAGE) using *β*-mercaptoethanol (1% w/v) to reduce the disulfide bridges. Finally gels were stained with Coomassie Blue G250.

### 2.2. Prediction of Bioactive Peptides from Nut Storage Proteins

*Carya illinoinensis* nut protein sequences were obtained from the GenBank data (https://www.ncbi.nlm.nih.gov/) [23] and analyzed to obtain the profile of cryptic peptides with putative functional activity using the BIOPEP database (http://www.uwm.edu.pl/biochemia/) [24]. Nut protein sequences for analysis in silico are as follows: Glutelin (44aa, superfamily Glutelin, GenBank accession: AAC69515.1), 11S legumin (505aa, superfamily Globulin, GenBank accession: ABW86979.1), 7S vicilin (784aa, superfamily Globulin, GenBank accession: ABV49593.1), 7S vicilin (792aa, superfamily Globulin, GenBank accession: ABV49592.1), putative allergen I1 (143aa, superfamily Albumin, GenBank accession: AAO32314.1), and putative 7S vicilin (102aa, superfamily Globulin, GenBank accession: AAZ93628.1). Occurrence frequency (*A*) of a bioactive fragment in a protein was given by the equation: *A* = *a*/*N*, where *a* is number of fragments with a given activity and *N* is number of amino acid residues [25].

### 2.3. Identification of Bioactive Peptides in the Nut Protein Fractions by Mass Spectrometry (MS/MS)

Nut protein fractions were digested with trypsin at a 1 : 50 ratio (trypsin : protein) for 16 h at 37°C and peptides were precipitated to obtain 200 mg of protein. Pellets were resuspended in 200 *μ*L of urea buffer (6 M Urea, 50 mM Tris-HCl pH 8), reduced with DTT at room temperature and alkylated with iodoacetamide in the dark. Reactions were stopped adjusting pH from 3 to 4 with formic acid [26]. The analysis of liquid chromatography-mass spectrometry was performed on a SYNAP-nanoUPLC System (Waters Co., Palo Alto, CA) equipped with an ionization source unit ion spray mass spectrometry. Peptide identification was performed using MASCOT (Matrix Sciences, http://www.matrixscience.com/) [27], with information obtained in BIOPEP database.

### 2.4. Determination of Antiproliferative Effect of the Encrypted Peptides of Nut Glutelins in Cell Lines

HeLa and CasKi cells (immortalized cells from cervical cancer) were grown in Dulbecco's modified Eagle's media (DMEM), with high concentration in glucose, and supplemented with NaHCO_3_ (3.5 mg/L), 10% fetal bovine serum, penicillin G (50 mg/L), streptomycin (100 mg/L), and amphotericin (1 mg/L), at 36°C and 10% of CO_2_. Apoptosis assay and cell cycle distribution were done using a FACS (fluorescence activated cell sorting) in apparatus Calibur (Becton Dickinson, NJ) [28]. HeLa and CasKi cells with a confluence of 85% were treated with 50 *μ*g/mL of trypsin-digested glutelins of nut. Cisplatin (1 *μ*g/mL) was used as a positive control of apoptosis. Primary culture of fibroblasts was used as the control of normal cells.

## 3. Results

### 3.1. Characterization of Storage Protein in Pecan Nut

**Figure 1:**
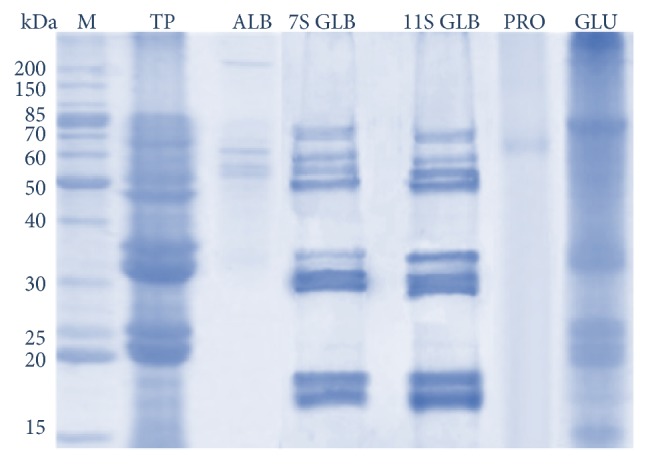
SDS-PAGE of nut's protein fraction. M, molecular weight marker; TP, total protein; ALB, albumins; 7S GLB, 7S globulins; 11S GLB, 11S globulins; PRO, prolamins; GLU, glutelins.

Pecan nut contained ~13.43 mg of total protein in 100 g of full-fat flour. Four soluble fractions were identified in nut total protein: albumins, globulins, prolamins, and glutelins. This last protein fraction was the most abundant representing ~53% followed by globulins, prolamins, and albumins, with percentages of ~27, 14, and 6%, respectively (Table 1). Fractions were separated by SDS-PAGE, detecting different protein patterns. For example, in the albumin fraction the proteins with apparent molecular weight of 220, 60, 55, and 52 kDa were observed. With 7S globulins the proteins of ~70, 60, 55, 50, 35, 32, 28, 18, and 17 kDa were visualized, whereas with 11S globulins the proteins with apparent molecular weight of 70, 60, 55, 50, 35, 30, 27, 20, 18, and 17 kDa were obtained. Under the conditions used in the assays, only one protein of ~65 kDa was resolved in the prolamin fraction, whereas in the glutelin fraction proteins of ~80, 35, 25, 22, 20, and 15 kDa were resolved (Figure 1).

### 3.2. Prediction of Bioactive Peptides Nut Storage Proteins

**Figure 2:**
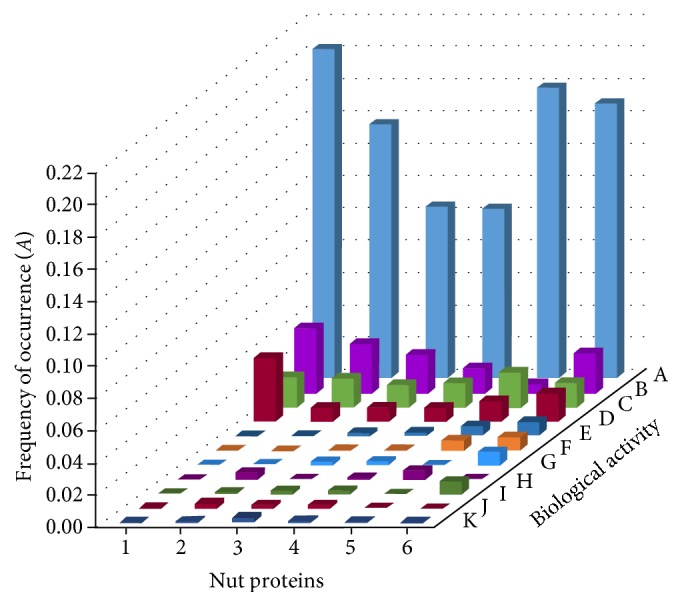
Prediction of peptides with potential biological activity in nut storage proteins with their occurrence frequencies. Nut proteins: (1) Glutelin (44aa, superfamily Glutelin), (2) 11S legumin (505aa, superfamily Globulin), (3) 7S vicilin (784aa, superfamily Globulin), (4) 7S vicilin (792aa, superfamily Globulin), (5) Putative allergen I1 (143aa, superfamily Albumin), and (6) Putative 7S vicilin (102aa, superfamily Globulin). Biological activity: (A) angiotensin I-converting enzyme-inhibitory activity (antihypertensive), (B) antioxidant (C) protease inhibitor, (D) metabolic stimulant, (E) metabolic regulator, (F) neuropeptide, (G) antithrombotic, (H) hypotensive, (I) antianesthetic, (J) activation of ubiquitin-mediated proteolysis (AUMP), and (K) immunomodulant.

The biological activities of putative peptides associated with nut storage proteins was predicted by bioinformatic analysis. Angiotensin I-converting enzyme-inhibitory activity (antihypertensive activity) had the higher occurrence frequency, followed by the antioxidant activity. Angiotensin I-converting enzyme-inhibitory activity was mainly found in glutelin (occurrence frequency 0.2045), followed by albumin (0.175) and three 7S globulins (0.1706) (Figure 2). In addition, the highest occurrence frequency for antioxidant activity was observed in glutelin (0.04) and 11S globulin (0.017), which are the most abundant fractions of nut (Table 1). A low occurrence frequency (<0.017) was observed for other nut storage proteins, with respect to glutelin and 11S globulin.

### 3.3. Peptides Identified in Tryptic-Digested Nut Storage Proteins by LC-MS/MS

LC-MS/MS analysis of nut storage proteins digested with trypsin showed 29 de novo peptides. In silico analysis showed that those peptides (Table 2) have putative biological activities, including antihypertensive, antioxidants, inhibitor of cell cycle (anticarcinogenic), antithrombotic, opioid, inhibitor of enzymes, activator of proteolysis, metabolic stimulant, and metabolic regulator. It has been reported that biologically active peptides are formed mostly by residues tyrosine, leucine, proline, glutamic acid, arginine, and alanine [3], and amino acids that were found in peptides of nut resolved by mass spectrometry analysis. As shown previously, the most abundant fraction in nut was glutelin. A total of 21 peptides with different biological activities were found: (a) in the glutelin fraction, peptides with antihypertensive, antioxidant, and anticarcinogenic activities were detected. (b) In the globulins fraction, peptide with antithrombotic activity that might inhibit the platelet aggregation was found. (c) When the prolamin fraction was analyzed, only peptides with opioid activity were observed. (d) In the albumin fraction, peptides with antihypertensive, antioxidant, and protease inhibitor activities were predicted. From all the biological activities predicted with the amino acid sequences of the peptides resolved by mass spectrometry, the antioxidant and antihypertensive activities showed the higher occurrence frequency, with values of 0.0821 and 0.063, respectively (Table 2).

### 3.4. Determination of Antiproliferative Effect of the Encrypted Peptides of Nut Glutelins in Cell Lines HeLa and CasKi

**Figure 3:**
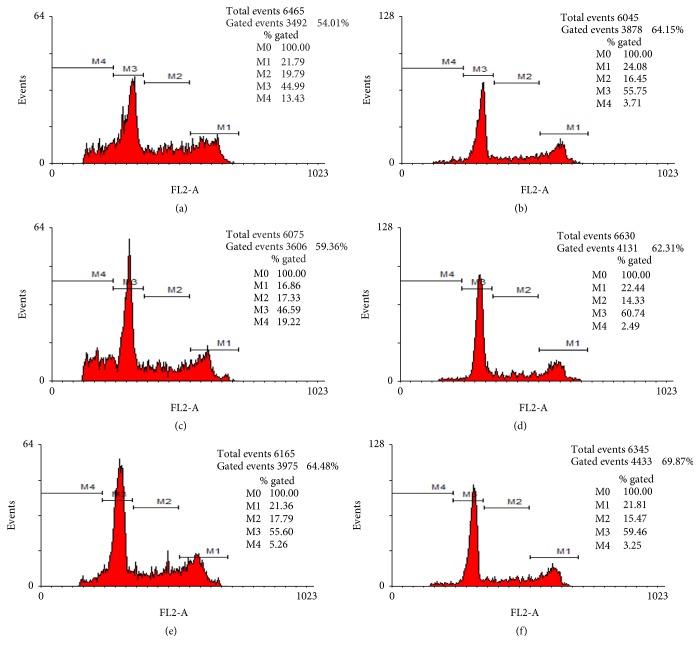
Cell cycle and antiproliferative effect of nut biopeptides on HeLa and CasKi cell lines. (a) HeLa cells treated with tryptic digest of nut, (b) untreated HeLa cells, (c) CasKi cells treated with tryptic sample, (d) untreated CasKi cells, (e) fibroblasts treated with tryptic digest of nut, and (f) untreated fibroblasts. M1, mitosis; M2, synthesis; M3, G_0_ phase; M4, apoptosis.

When HeLa cells were treated for 36 h with 50 *μ*g/mL of tryptic digests of nut, it is observed that 44.99%, 19.79%, 21.79%, and 13.45% of cells were in G_0_ phase, synthesis, mitosis, and apoptosis, respectively, while untreated HeLa cells showed that the 55.75% was in phase G_0_, 24.08% in mitosis, and 3.71% in apoptosis. CasKi cells treated with tryptic digests showed that 46.59%, 17.33%, 16.83%, and 19.22% were in G_0_ phase, synthesis, mitosis, and apoptosis, respectively, whereas untreated CasKi cells showed that 60.74% were in the G_0_ phase, 14.33% in synthesis, 22.44% in mitosis, and 2.49% in apoptosis. Interestingly, when this activity was analyzed in healthy control cells (fibroblasts) treated in the same way as the cervical cancer cell, we found that 55.60% were in G_0_ phase, 17.79% in synthesis, 21.86% in mitosis, and 5.26% in apoptosis, whereas in the untreated fibroblasts 59.46%, 15.47%, 21.81%, and 3.25% of the cells were G_0_ phase, synthesis, mitosis, and apoptosis, respectively, which showed that antiproliferative activity can be selective of the neoplastic cells (Figure 3).

## 4. Discussion

There is special interest to know the nutritional or biological properties of walnut, mainly because people are using it as a common fruit in their diet, eating it alone as a dried fruit or in different food products such as desserts. To our knowledge no information has been published about encrypted peptides from nut proteins, which can be released during the normal process of digestion and produce beneficial physiological effects.

Firstly, we determined soluble protein fraction in nut. It was found that this fraction is formed by albumins, globulins, prolamins, and glutelins, which have been also reported in plants such as amaranth. The highest soluble fraction was represented by glutelins with ~53.27%. This percentage was higher than that reported in amaranth (21–24%) but lower than those found in wheat and melon (85 and 82%, resp.) [29, 30]. The concentration of 11S globulins was slightly lower than that found in amaranth [31]. In spite of the fact that we used modified protocols for extracting prolamin (i.e., ethanol plus sodium acetate and *β*-mercaptoethanol) [20, 22, 26], we only were able to obtain ~14% of the total fractions.

Bioinformatic analysis predicted that angiotensin I-converting enzyme-inhibitory activity, which is related to antihypertensive, was the main biological activity found in nut storage proteins followed by the antioxidant effect. This is an important finding as the nut consumers might be protected against hypertension problems and also ingest molecules with antioxidant activity. It is known that angiotensin I-converting enzyme (ACE) (EC 3.4.15.1) is a hypertension-responsible glycoprotein present both in biological fluids and in many tissues [32]. This enzyme can be inhibited by small encrypted peptides, for example, LKPNM and LKP, originating from fish proteins, are able to inhibit the ACE and showed activities of 66 and 91%, respectively, compared with the captopril [33]. Also, it has been confirmed that different peptides derived from protein eggs showed ACE inhibitory effect and antihypertensive effect [34]. It will be interesting in future studies to synthesize some of the peptides found in the glutelin and globulin fractions of nut (i.e., DMIPAQ, EEE, LKAWSVAR, VISR, LAASGLLLL, ALLALS, VDG, FQP, and CYFQNCPR) (Table 2) and test their potential to have an ACE inhibitory activity. On the other hand, although the antioxidant activity in nut is mainly associated with the presence of phenolic compounds [32], the occurrence frequency of the antioxidant activity owing to encrypted peptides suggest that its antioxidant effect could be not only for the presence of the phenolic compounds but also for encrypted peptides. These peptides might act as chelators of metal ions, thereby preventing cellular oxidation [35, 36].

Another activity resulting in relatively high values in the frequency of occurrence was the activity as protease inhibitor (0.002) found in albumin fraction. Peptide protease inhibitors produced by the plants are small amino acids that contribute to the defense against insects [37] and they are found primarily in grains and storage tissues of plants. Additionally and according to our in silico analysis, it seems that albumin fraction contains encrypted peptides that inhibit the dipeptidyl aminopeptidase IV (DPP-IV) (EC 3.4.14.5) and activate the ubiquitin-mediated proteolysis. This finding is important because accelerating the uptake of encrypted peptides, by activating a ubiquitin-dependent proteolytic pathway, can reduce the blood glucose level in human being [16, 38]. Additionally, we found that that globulin and prolamins fractions may have antithrombotic and opioid peptides, respectively, which can act as platelet aggregation inhibitors and powerful painkiller [14, 39].

We predicted that glutelin fractions might have an anticarcinogenic activity, and we demonstrated experimentally that protein fractions of nut digested with trypsin has an antiproliferative effect on HeLa and CasKi cell lines derivated of cervical cancer. We detected the presence of peptides AYRNRYRRQYRY, EQRPRT, and LPTSEAAKY in glutelin fraction, which might be responsible for the antiproliferative effect. Although we do not have experimental data, it is possible those last two peptides might play an important role in the inhibition of the transcription factor STAT3, which mediates the expression of various genes involved in cell proliferation and apoptosis [12]. In conclusion, we demonstrated that digested tryptic protein samples, which contain encrypted peptides, are able to exert an antiproliferative activity on neoplastic cells. Our aim in future studies will be focused in testing the different biological activities using synthetic peptides with the amino acid sequences reported here.

## Figures and Tables

**Table 1:** Pecan nut storage proteins quantification.

Protein	Storage protein fraction	Nut flour (mg/g)	Percent (%)
Soluble	Albumin	1.97^a/a*∗*^ ± 0.04	5.80^a/b^
	7S Globulin	0.95^b/a^ ± 0.03	2.80^b/b^
	11S Globulin	8.09^c/a^ ± 0.03	23.83^b/b^
	Prolamin	4.85^d/a^ ± 0.19	14.28^c/b^
	Glutelin	18.08^e/a^ ± 0.30	53.27^d/b^

Insoluble		22.00 ± 2.43	

Total		291.92 ± 11.64	

^*∗*^Means with different superscript letter indicates significant difference (*p* < 0.05).

**Table 2:** Biopeptides identified in the tryptic digest of fractions of Pecan nut storage proteins by spectrometry MS/MS.

Activity	Occurrence frequency	Peptides with biological activity	Storage protein	Description and report	Reference
Antihypertensive	0.063	MVISR, LAASGLLLL, ALLALS, VDG, FQP, DMIPAQ, EEE, LKAWSVAR	Glutelins	ACE I inhibitor	[10]
		CYFQNCPR	Albumins and Glutelins		

Antioxidant	0.0821	GYY, Ell, IRWH, TFEEETSA, NYKQMT	Glutelins	It protects the cell from oxidation	[11]
		YYY, LKPPTY, YYG, LEGFYYY	Albumins and Glutelins		

Antiproliferative (anticarcinogenic)	0.031	AYRNRYRRQYRY	Glutelins	Inhibitor of oncogenic transcription factor (STAT3)	[12]
		EQRPRT, LPTSEAAKY			

Antithrombotic	0.0016	DEE	11S Globulins	Antiplatelet	[13]

Opioid	0.0091	YPFPGPIP, GYK, QK	Prolamins	Powerful Painkiller	[14]

Protease inhibitor	0.002	LA	Albumins	Inhibitor of dipeptidyl aminopeptidase IV	[15]

Activators of ubiquitin-mediated proteolysis	0.00843	LA	Albumins	Activator of ubiquitin-mediated proteolysis	[16]

Other functions (metabolic stimulants and metabolic regulator)	0.001	NPHDHQ, LEANPRS, WLTTIHGS	Albumins and glutelins	—	[10]
